# Leucine-rich repeat protein PRAME: expression, potential functions and clinical implications for leukaemia

**DOI:** 10.1186/1476-4598-9-226

**Published:** 2010-08-27

**Authors:** Frances Wadelin, Joel Fulton, Paul A McEwan, Keith A Spriggs, Jonas Emsley, David M Heery

**Affiliations:** 1 Gene Regulation Group, Centre for Biomolecular Sciences, School of Pharmacy, University of Nottingham, Nottingham NG7 2RD, UK; 2 Protein Structure Group, Centre for Biomolecular Sciences, School of Pharmacy, University of Nottingham, Nottingham NG7 2RD, UK; 3 RNA Biology Group, Centre for Biomolecular Sciences, School of Pharmacy, University of Nottingham, Nottingham NG7 2RD, UK

## Abstract

PRAME/MAPE/OIP4 is a germinal tissue-specific gene that is also expressed at high levels in haematological malignancies and solid tumours. The physiological functions of PRAME in normal and tumour cells are unknown, although a role in the regulation of retinoic acid signalling has been proposed. Sequence homology and structural predictions suggest that PRAME is related to the leucine-rich repeat (LRR) family of proteins, which have diverse functions. Here we review the current knowledge of the structure/function of PRAME and its relevance in leukaemia.

## PRAME is a cancer-testis antigen

PRAME, or preferentially expressed antigen in melanoma, was originally identified as a gene encoding a HLA-A24 restricted antigenic peptide presented to autologous tumour-specific cytotoxic T lymphocytes derived from a patient with melanoma [1]. PRAME is synonymous with MAPE (melanoma antigen preferentially expressed in tumours) and OIP4 (OPA-interacting protein 4), and its expression profile defines it as a cancer-testis antigen [1]. Cancer-testis antigens (CTAs) are encoded by non-mutated genes expressed at high levels in germinal tissues and tumours, but which are absent from or detected at low levels in other tissues [2]. Examples include the MAGE, BAGE, GAGE and MAPE/PRAME protein families, all of which have been detected in tumours of many different histological types [2]. PRAME may be somewhat different to other cancer-testis antigens in that it shows some expression in normal tissues such as ovary, adrenal, placenta and endometrium [1]. The C-terminus of human PRAME (amino acids 453-509) was also identified in a yeast two-hybrid screen for host cell proteins that bind *Neisseria gonorrhoeae *opacity factors, in this case the OPA-P protein [3]. Thus PRAME is also known as OIP4 (OPA interacting protein), although the functional implications of the interaction are unknown. Interestingly, another cancer-testis antigen (OIP5) was isolated in the same screen [4].

## Gene structure, expression and transcripts

**Figure 1:**
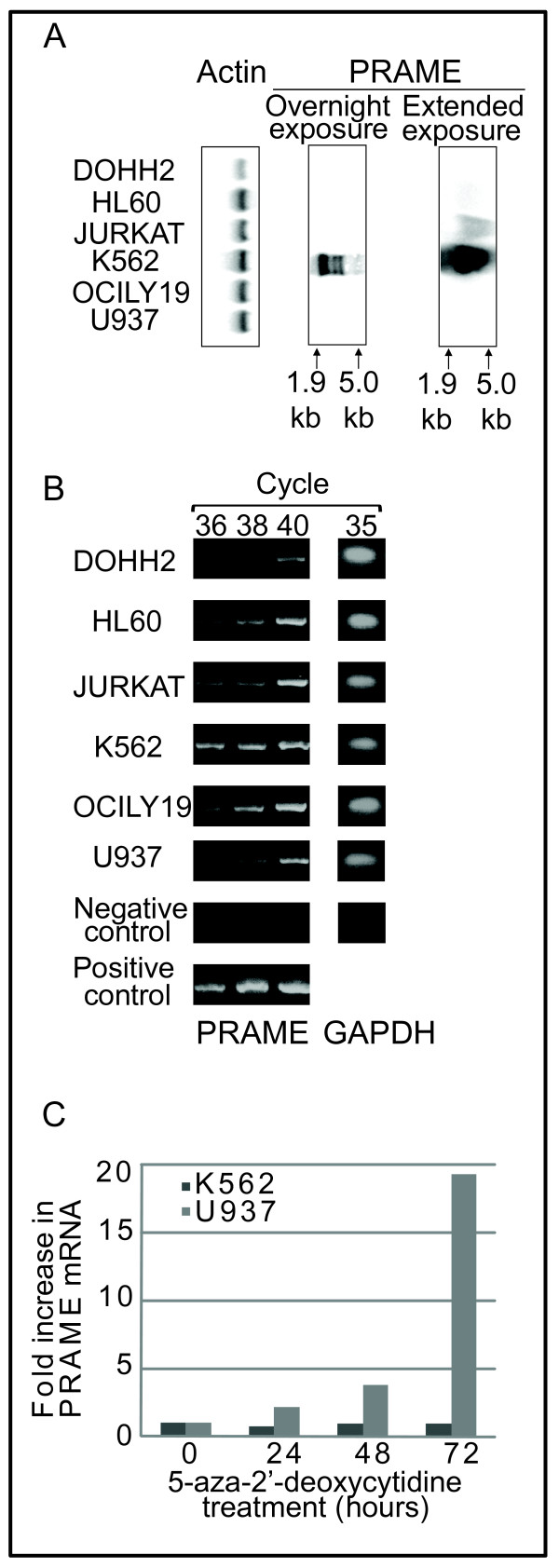
**PRAME expression in leukaemia and lymphoma cell lines**. A: Northern blot analysis of PRAME expression in leukaemia and lymphoma cell lines. Samples contained 50 μg of total RNA extracted from tumour cell lines. After Northern blotting, membranes were hybridised with a ^32^P-labelled probe consisting of full-length PRAME coding region, washed at high stringency and visualised using a phosphorimager. A control probe (β-actin) was used to confirm equal loading. Both overnight and extended exposures are shown. B: Semi-quantitative RT-PCR analysis of PRAME and GAPDH expression in leukaemia and lymphoma cell lines. RNA was extracted and reversed transcribed using oligo(dT)_12-18_. cDNA was amplified using primers: PRAME forward (5'atggaacgaaggcgtttg-3'), PRAME reverse (5'-ctagttaggcatgaaacaggg-3'), GAPDH forward (5'-aggtgaaggtcggagtcaac-3') and GAPDH reverse (5'-gatgacaagcttcccgttct-3'). An aliquot of the PCR reaction was removed after 36, 38 and 40 cycles for the PRAME reaction as indicated, or 35 cycles for the GAPDH control. PCR products were visualised by gel electrophoresis. C: Induced expression of PRAME in U937 after DNA demethylation. Leukaemia cell lines U937 (low levels of PRAME) and K562 (PRAME overexpressed) were cultured in RPMI plus 10% foetal bovine serum and treated with 1 μM 5-aza-2'-deoxycytidine for 0-72 hours. RNA was extracted and reverse transcribed for expression analysis. PRAME mRNA levels were quantified by real-time qPCR using the following primers: PRAME 254F (tgctgatgaagggacaacat), PRAME 364R (cagcacttgaagtttccacct). GAPDH primers were as in Fig. 1B. Fold increase in PRAME expression was calculated by the standard delta-delta CT method, relative to GAPDH.

The human PRAME gene is encoded on the reverse strand of chromosome 22 (22q11.22) extending over a region of approximately 12 kilobases. It is located within the human immunoglobulin lambda gene locus [5] which contains a large number of V_λ _gene segments used to generate λ light chains during B cell development. This locus also contains several other non-immunoglobulin genes, for example PRAME is situated between tandem Suppressor of Hairy Wing genes (SUHW1/ZNF280A and SUHW2/ZNF280B) and a gene encoding a putative membrane glycoprotein (POM121L1). Adjacent to POM121L1 is the pseudogene BCR4 (or BCR4L), which has been identified as a breakpoint cluster region implicated in chromosome 22 rearrangements [6]. BCR4 shows significant homology to the 3' end of the original BCR gene at the Philadelphia chromosome breakpoint [6]. Interestingly, the BCR4 region is known to be amplified in the CML-derived cell line K562 [6]. Consistent with this, Northern blots (Fig. 1A) and semi-quantitative PCR data (Fig. 1B) confirmed that PRAME mRNA is highly elevated in K562 cells in comparison to other cell types such as Jurkat, U937 and HL60 which express PRAME at lower levels [1].

A number of PRAME mRNA transcripts showing differential abundance have been detected in normal testis, malignant tissues and leukaemia-derived cell lines [1,7]. The NCBI database annotates five PRAME mRNA transcripts ranging from 2.1-2.7 kb in length (2141, 2162, 2197, 2220, 2776 bases) and a qPCR study of these 5 mRNAs reported that the two shortest transcripts were the most abundantly expressed in testis and leukaemia cell lines [7]. However, sequence databases list at least 17 different PRAME mRNAs, the largest of which is a 3329 base transcript that is clearly detectable in northern blots of total RNA isolated from various cancer cell lines such as K562, Hela and HL60 (Fig. 1A and reference 1). Each of the major transcripts contains 6 exons, four of which contain coding sequence, and all encode an identical polypeptide of 509 amino acids. Differences in the 5' ends of these transcripts suggest the existence of alternative transcription start sites. This is further supported by the strong promoter activity in reporter assays displayed by the sequence around the proximal transcription start site including exon 1a and the first intron of the PRAME gene (-165 to +365) [7].

Expressed sequence tags suggest that alternative splicing may produce up to 15 splice variants of PRAME, some of which potentially encode tissue-specific truncated PRAME proteins, although this remains to be verified by western blotting. A polyclonal antibody against PRAME raised by the Coulie group [8] and a commercial antibody (Abcam 32185) used in 2 studies [9,10], recognise a protein of the expected size (approximately 58kDa) in PRAME-expressing cell lines, although an apparently non-specific cross-reacting protein is also detected at around 75kDa [9]. There is a report in the literature of a monoclonal antibody specific for PRAME, although the protein detected (33kda) in CLL cells was below the expected size for full length PRAME [11]. Whether this corresponds to a truncated variant of PRAME in CLL or interaction with a PRAME-like protein remains unclear.

Four of the five validated PRAME transcripts contain unique 5' untranslated regions (5' UTRs). Similar sequence diversity is observed in the 5'UTRs of PRAME transcripts in other primates, suggesting these sequences may have functional significance in regulating PRAME expression in response to metabolic or developmental signals. As most PRAME 5' UTRs contain multiple start and stop codons they would be expected to inhibit productive protein synthesis under the canonical model of cap-dependent translation. In agreement with this, RNA structure prediction algorithms indicate that human PRAME 5' UTRs can form stable secondary structures, a feature likely to inhibit translation initiation under normal conditions. Indeed, data from our group indicates that PRAME translation is inhibited by hippuristanol (an inhibitor of the RNA helicase eIF4A required for translation of structured mRNAs) [FW, KS & DMH; unpublished].

## Regulation of PRAME and expression in malignancies

While PRAME is absent or expressed at very low levels in most normal tissues tested, high levels of PRAME mRNAs are encountered in malignant cells, including the vast majority of primary and metastatic melanomas (88% and 95% respectively) [2]. Microarray and PCR studies have shown that PRAME is absent in normal haematopoietic tissues including bone marrow, CD34+ sorted bone marrow cells, unsorted peripheral blood cells and sorted B and T lymphocytes [12-15]. However, numerous studies have reported highly elevated levels of PRAME in both acute and chronic leukaemias and non-Hodgkin's lymphomas (Fig. 1B and references 12,13,15-19). PRAME up-regulation was observed in most AML cases with t(8;21) karyotype and 45% of AML cases with t(15;17) [13,16,17]. Significant association of PRAME expression has also been reported in ALL (17-42%) [18,19], CLL (27%) [11,20], myeloma (23-52%) [21,22] and chronic phase CML (36%) [12]. In CML, PRAME expression was found to correlate with disease progression, showing increased expression in blast crisis as compared with chronic phase disease [12,15]. PRAME is also associated with solid organ cancers including skin [1], breast [23-25], lung [1], head and neck cancers [26] and neurological neoplasms [27,28].

The regulation of PRAME gene expression is poorly understood, and thus the molecular basis of its expression in malignancies is largely unknown. It has been suggested that AML1-ETO and BCR-ABL fusion proteins may contribute to the up-regulation of PRAME [13,29], whereas SOX9 has been reported to repress PRAME expression [30]. However, no correlation was observed between expression of PRAME and SOX9 in samples from patients with CML [15]. Like other cancer-testis antigens [31,32], the PRAME gene is hypermethylated in normal tissues such as bone marrow, but hypomethylated in malignant cells [7,33-35]. As a consequence, in cell lines such as U937 that show low level PRAME expression, treatment with DNA demethylators such as 5'-aza-2'-deoxycytidine can strongly induce PRAME transcription (Fig. 1C) and studies have shown that this correlates with demethylation of specific cytosine/guanine dinucleotide rich regions in the PRAME promoter [7,33,34]. Similarly, methylation-specific PCR analyses demonstrated that hypomethylation of the PRAME promoter is significantly more frequent in CML with blast crisis compared with chronic phase disease [34]. PRAME mRNA is found frequently in advanced stages of malignancies such as melanoma [2], neuroblastoma [28] and breast cancer [23], though not in the earlier stages of these diseases. Thus, PRAME may have a role in disease progression, although whether it is a driver or passenger gene remains to be established.

Interestingly, a recent comparative genomic hybridisation study detected microdeletions in the lambda immunoglobulin light chain locus (22q11) in 18% of untreated CLL cases, and also in cases of acute promyelocytic leukaemia and non-Hodgkin's lymphoma [36]. The minimally deleted region included the ZNF280A, ZNF280B and PRAME genes and both mono-allelic and bi-allelic deletions were observed. In some cases the deletion of the 22q11 locus was the sole chromosomal abnormality detected, suggesting it may be an important factor in disease pathogenesis. Absence of PRAME expression in this CLL subgroup was confirmed by qPCR, but did not correlate significantly with other clinico-pathological factors, although a significant correlation was found for expression of lambda surface light chain in 22q11 deletions [36]. Homozygous deletion of this region (22q11.22) was also reported in cases of mantle cell lymphoma and corresponding cell lines, and the absence of both PRAME and ZNF280A expression was confirmed [37]. It remains to be established whether loss of PRAME or ZNF280A genes are significant contributory factors in the pathogenesis of leukaemias and non-Hodgkin's lymphomas.

## The PRAME multigene family

PRAME is a member of a multigene family present in humans and other mammals. However, orthologous genes appear to be absent in fish, amphibians and invertebrates. Like several other cancer-testis antigen gene families, PRAME appears to have undergone multiple gene duplications during hominid evolution, and at least 22 PRAME-like genes and 10 pseudogenes have been identified in the human genome [38]. This rapid evolution is consistent with adaptive (positive) selection similar to gene clusters involved in immunity and reproduction, such as the NALP family [39]. Interestingly, several PRAME-like proteins and NALPs have been proposed to be involved in gametogenesis, folliculogenesis and early embryogenesis in the mouse [40]. Oogenesins 1-3 are PRAME-like proteins that show highly selective expression in mouse ovary, whereas Oogenesin 4 was detected in both ovary and testis [41]. These proteins show considerable homology to PRAME and PRAME family members.

## PRAME is a leucine rich repeat (LRR) protein

**Figure 2:**
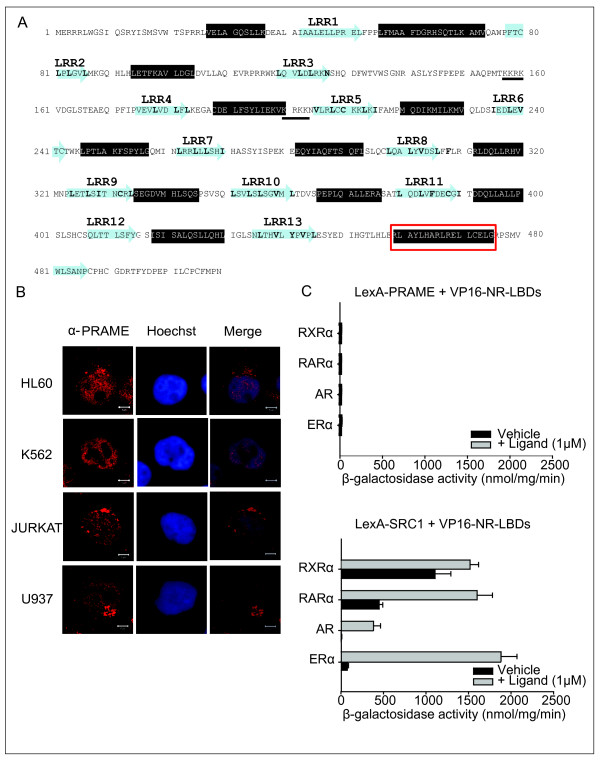
**PRAME LRR repeats, subcellular localisation and interaction with nuclear receptors**. A: Predicted domain structure of the human PRAME sequence indicating potential Leucine Rich Repeats (LRRs). The LRRs are numbered and indicated by the blue arrows; residues conserved in typical LRRs are highlighted in bold. The black boxes indicate regions predicted to have a high probability of α-helicity, and two predicted NLS sequences are underlined. The boxed area in red is a region implicated in interaction with retinoic acid receptors, and potentially contains LXXLL and CoRNR box-like motifs. B: Subcellular localisation of endogenous PRAME proteins in leukaemia cell lines. Leukaemia cell lines were cultured as described in the legend to Fig. 1, and harvested onto coverslips using a cytospin centrifuge. Cells were fixed in 4% paraformaldehyde, permalised with 0.2% Triton X-100 and blocked with 3% PBS prior to application of an α-PRAME antibody (Abcam ab31285) followed by secondary antibody (Alexa Fluor 594 chicken anti-rabbit IgG - Invitrogen A21442). DNA was stained with a Hoechst stain (Sigma Aldrich 332581). Images were captured using LSM 510 Meta confocal laser scanning microscope (Zeiss). C: Yeast two-hybrid experiments to assess interactions of SRC1 nuclear receptor interaction domain (431-761) or full-length PRAME (1-509) with nuclear receptors were performed using the reporter strain *S.cerevisiae *L40 as described previously [68,69]. PRAME and SRC1 domains were expressed as LexA fusion proteins. Nuclear receptor ligand binding domains (RARα 200-464; RXRα 230-467; ERα 282-595; AR 625-919) were expressed as VP16 activation domain (411-490) fusion proteins, and reporter (β-galactosidase) specific activity was determined as described previously [68,69].

PRAME is a leucine-rich protein of which 21.8% of residues are leucine or isoleucine. Homology searches reveal that PRAME and PRAME-like proteins contain leucine-rich repeats (LRR), and are thus related to the LRR containing protein family (Fig. 2A) [42]. Typical LRR motifs such as those present in ribonuclease inhibitors are 20-30 amino acids in length, and contain the consensus sequence LXXLXLXXN/CX_(1/2)_L [43]. The LRR repeat forms a beta sheet followed by an α-helix, and the repeating units can induce a curved solenoid (horseshoe) fold with a parallel beta sheet on the concave side and helical elements on the convex side [44]. However, not all LRRs fit this consensus and atypical repeats are found among some families including the PRAME and NALP families. Secondary and tertiary structure predictions e.g. using Phyre software [45] suggest that PRAME is likely to adopt a fold similar to the LRR domains of Toll-like receptors (TLR3, TLR4) and internalin proteins (reference [38] and our unpublished analyses). The tertiary structure of the LRR stack provides an ideal module for molecular interactions with proteins, nucleic acids and other ligands; and LRR domains have important functions in cell immunity, cell adhesion and signal transduction. For example, in cell membrane-associated TLR proteins the LRR moiety is extracellular, and functions in sensing pathogen-associated molecular patterns (PAMPs) [46]. Intracellular LRR proteins, such as NALP family, are also likely to be activated by PAMPs in antimicrobial immune responses, resulting in regulation of inflammation and apoptosis pathways [46]. Thus, the interaction of PRAME with a bacterial pathogenicity protein is intriguing, although, it remains to be established whether PRAME plays a role in immune response pathways.

## Subcellular localisation of PRAME

The subcellular localisation of PRAME has been examined in a number of studies using different cell lines to express recombinant epitope-tagged or GFP-tagged PRAME. Over-expression of PRAME-FLAG and PRAME-GFP proteins in CHO cells (which do not express PRAME) was reported to induce aberrant cell morphology and cell death. However, in transfected cells expressing low levels of the PRAME-GFP, the protein was observed to localise to both the nucleus and perinuclear regions [47]. Consistent with this, we have detected PRAME-GFP and PRAME-FLAG in nuclear and cytoplasmic compartments, dependent on the cell type. For example, PRAME-GFP was found both in the nucleus and cytoplasm of Hela cells, whereas it was observed to be mainly cytoplasmic in U2OS cells (data not shown). In addition, in leukaemic cell lines that express high levels of the native protein, PRAME appears to be detected both in the nucleus and the cytoplasm (Fig. 2B). Consistent with its ability to localise to the nucleus, PRAME contains several candidate nuclear localisation signal (NLS) sequences including 157-KKRKV-161 and 198-KVKRKKNV-205 (Fig. 2A)

## Cellular functions of PRAME: repression of retinoic acid receptor signalling

PRAME has been reported to function as a repressor of retinoic acid (RA) signalling through interactions with retinoic acid receptors (RARs) and repression of the RARβ2 gene [8,48]. RARs are important regulators of haematopoietic differentiation and apoptosis. In the absence of ligand, RARs can repress their target genes by recruitment of SMRT and NCOR co-repressor complexes, which have associated histone deacetylase (HDAC) activities. Binding of retinoic acid induces a change in the conformation of the RAR ligand binding domain (LBD), promoting the recruitment of co-activator complexes with histone acetyltransferase activities. This can promote transcription of RA target genes, regulating differentiation, cell cycle arrest and caspase-dependent apoptosis pathways in responsive cells. The RARβ2 gene is highly up-regulated by RA, and is believed to be responsible for many of the beneficial effects of RA in cancer cells. As a consequence, suppression of RA responsiveness through hypermethylation of the RARβ2 gene promoter, which is a common feature of tumours, supports the hypothesis that RARβ2 has important tumour suppressor functions. Thus, it was proposed that repression of RAR function by PRAME might be an important contributory factor in AML disease progression [8].

Epping and colleagues demonstrated that over-expressed TAP-tagged PRAME can be co-immunoprecipitated with RARα, with the interaction being dependent on the C-terminus of PRAME [8]. Weak direct interactions of PRAME and RARα in GST-pulldown experiments were reported, whereas no binding of PRAME to ER or RXR was detected [8]. Due to its leucine-rich content, PRAME contains at least seven sequences matching the consensus of the LXXLL signature motif found in many nuclear receptor binding proteins [49]. However, only one of these (i.e., 467-LRELLCE-473, located close to the C-terminus of PRAME) was found to contribute to interactions with the RARα LBD *in vitro *[8]. Unlike most other LXXLL motif containing cofactors, the interaction of PRAME with RARα was not reported to be dependent on ligand. Moreover, mutations in the RARα AF2 helix, which is essential to generate the LXXLL peptide binding surface, did not alter the ability of PRAME to repress RA signalling [48].

Nuclear receptor co-repressors such as SMRT and NCoR contain CoRNR box motifs, which have the consensus of LXXX(I/L)XXX(I/L). These motifs mediate ligand-independent binding to nuclear receptors. Two of the seven motifs in PRAME, including the most C-terminal motif, also fit the CoRNR box consensus (463-LHARLRELLCELG-475). Thus, it remains to be tested whether mutations in the RAR LBD that disrupt binding to CoRNR box peptides would also disrupt the ability of PRAME to impact on RA signalling. However, until the structure of the PRAME protein is known, it remains unclear, whether any of these motifs are available for protein-protein interactions. In our hands, the binding of PRAME to the LBDs of RARα or other nuclear receptors in GST pulldown (data not shown) or yeast two-hybrid assays (Fig. 2C) is very weak in comparison with other cofactors such as SRC1. Thus, the possibility remains that indirect interactions with other proteins may be important to facilitate the functional interactions of PRAME and RARs *in vivo*.

PRAME-mediated suppression of RA signalling was reported to involve the recruitment of the polycomb PRC2 complex component, EZH2. Over-expressed PRAME and EZH2 were shown to co-immunoprecipitate, although whether this is via direct interactions was not addressed [8]. Knockdown of EZH2 and EED relieved PRAME-dependent repression of the RARβ2 promoter, as did over-expression of a mutant of EZH2 defective in its SET domain methyltransferase function [8]. In addition to its reported interaction with RARs and EZH2, yeast two-hybrid screens have identified several other proteins that appear to interact with PRAME. However, the functional significance of reported interactions with nuclear bacterial OPA-P protein [3], the nuclear kinase STK19 [50], and UBE21 [51] remains to be investigated.

HDAC inhibitors (HDACi) can induce proliferation arrest and apoptosis of cancer cell lines, and have shown promise as anticancer agents in preclinical models and clinical trials. In a separate study, Epping and colleagues isolated both RAR and PRAME in a genetic screen for proteins that could block the effects of HDACi on cell proliferation [48]. It was shown that ectopic expression of PRAME blocked the suppressive action of HDACi compounds in colony formation assays. PRAME also blocked HDACi-mediated induction of RARβ2 and p21 genes, but had no impact on the effects of conventional chemotherapeutics (such as cisplatin, fluorouracil and bortezomib in the same model) [48].

The function of PRAME and its effect on gene expression in leukemic cells remains controversial due to conflicting observations in the literature. While in cell-based models PRAME was reported to down-regulate genes such as S100A4, RARβ2, p21 and Hsp27 [47], a clinical study reported that expression levels of these genes were not significantly associated with PRAME expression in paediatric AMLs [52]. A focussed microarray study of childhood AMLs examining the expression of 300 stress-related genes reported a correlation between PRAME expression with up-regulation of multidrug resistance genes (MRP3 and BCRP) and down-regulation of pro-apoptotic genes (CIAP2, AKT3, BAK1, BAX) [53]. However the same genes were unaffected when PRAME was over-expressed or silenced in cervical cancer cell lines [52]. Thus, further insight is needed into the tissue-specific effects of PRAME in gene regulation, and how this is achieved.

## Effects of PRAME on cell proliferation and differentiation

The role of PRAME in proliferation and differentiation of haematopoietic tissues appears complex. For example, PRAME expression was associated with reduced proliferation of KG-1 leukaemic cells [47]. In the same study, knockdown of PRAME caused significantly increased tumorigenicity of K562 cells in a xenograft model, which was suggested to be due to reactivation of pro-apoptotic genes in the absence of PRAME [47]. In a more recent study, over-expression of PRAME was found to promote proliferation of various leukaemic cell lines and inhibit ATRA (all-*trans *retinoic acid)-induced myeloid differentiation [15]. However, these effects were found to be cell line-specific as they were only observed in cell lines that undergo myeloid differentiation following ATRA exposure. When PRAME was over-expressed in normal haematopoietic progenitor cells in the presence or absence of ATRA, myeloid differentiation was inhibited, although proliferation appeared unaffected. Furthermore, shRNA silencing of PRAME in primary cells led to increased myeloid differentiation. Thus, the consequences of induced PRAME expression for proliferation and differentiation of haematopoietic cells appears to be dependent both on cell lineage, and contributing factors involving other genetic or epigenetic mechanisms.

## Clinical applications of PRAME: risk stratification, minimal residual disease monitoring and immunotherapy

Although the role of PRAME in acute leukaemia and other cancers is complex, it has promise both as a cancer biomarker and as a therapeutic target. In AML, PRAME is usually associated with a favourable response to chemotherapy and prolonged survival [13,14,54]. This was initially thought to be due to its expression in leukaemias having favourable prognoses, such as AML M2 with t(8;21), AML M3 with t(15;17) and childhood B-ALL [13,16,55]. However, PRAME has been reported to be an independent prognostic factor in AML M3 with t(15;17) [16] and to be associated with longer overall survival, even in karyotypes with generally poor prognosis such as deletion of the long arm of chromosome 7 and monosomy 7 [54]. In contrast, over-expression of PRAME mRNA is associated with poor prognosis in solid organ malignancies [23,24,28]. This raises the possibility that PRAME may have different roles in oncogenesis or tumour suppression dependent on the tumour type. Therefore, its usefulness in predicting clinical outcome in solid tumours remains unclear. However PRAME remains relevant in acute leukaemias for risk stratification, to monitor residual disease and as a potential target for immunotherapies.

Disease risk-stratification is imperative in order to best tailor therapies to meet a patient's individual needs. At least a third of patients with *de novo *AML have normal cytogenetics and therefore are difficult to risk stratify [56]. Thus attempts have been made to construct a molecular stratification model for this group of intermediate risk patients, which includes PRAME expression [57]. Low PRAME expression at diagnosis was found to be associated with disease refractory to induction chemotherapy, shorter relapse-free survival and poorer overall survival [57]. In cancers other than acute leukaemias, PRAME expression is associated with negative outcomes. Indeed, high PRAME expression is associated with increased resistance to common chemotherapeutic regimens in diffuse large B cell lymphomas and Hodgkin's disease [58,59]. Furthermore, it is associated with failure of second-line therapies and the development of point mutations in the ABL tyrosine kinase domain in chronic phase CML [15]. PRAME has also been proposed as a prognostic marker for poor outcome in solid tumours such as breast and ovarian cancers [10,23,24].

In addition, PRAME has been shown to be a useful indicator of minimal residual disease (MRD) in both acute and chronic leukaemias [16,18,55,60,61]. While PRAME mRNA was often significantly over-expressed in bone marrow samples from patients with newly diagnosed leukaemia (as compared with healthy donors), its expression level decreased to normal levels in patients who responded to treatment [17,18,60]. Moreover, increasing levels of PRAME mRNA have been detected in patients undergoing relapse, even prior to cytological diagnosis [16,18,60]. This is particularly advantageous in monitoring MRD in patients without known genetic markers.

CTAs were originally identified as proteins containing epitopes that induced cell-mediated immune responses in cancer patients. Thus they represent promising candidates for the development of immunotherapies specifically targeting cancer cells [62]. PRAME epitopes were reported to be recognised by HLA-A24 restricted cytotoxic T lymphocytes [1]. Thus, a specific immunotherapy targeting PRAME might offer a therapeutic benefit in graft versus leukaemia effects observed after allogeneic stem cell transplant, or to prolong a complete remission achieved by chemotherapy. PRAME has already been shown to be a prime candidate for such an immunotherapy, inducing strong immune responses in healthy volunteers and patients with AML, CML, ALL and melanoma [9,54,63,64], and could potentially form a polyvalent vaccine with other cancer-testis antigens [65,66]. Unfortunately, like other CTAs, PRAME can display heterogeneous expression levels within tumours, which could potentially allow some malignant cells to escape immunotherapy. Studies have shown that treatment with agents such as 5'-aza-2'-deoxycytidine [7,33-35] and clofarabine [67] can induce the expression of CTAs (including PRAME) through DNA demethylation. Thus, while anti-PRAME vaccines are currently being evaluated in clinical trials for their efficacy against PRAME-positive tumours, combination with demethylating agents to maximise CTA expression may be required for complete elimination of the tumour cells.

## Conclusions

**Figure 3:**
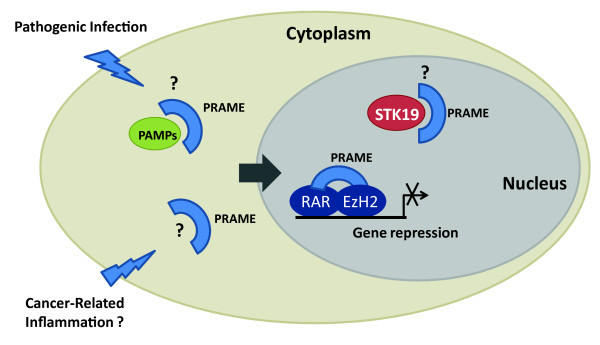
**Potential nuclear and cytoplasmic functions of PRAME**. Schematic representation depicting interactions of PRAME with nuclear proteins such as retinoic acid receptor (RAR), polycomb repressor EZH2 and the serine threonine kinase STK19. Interaction with RARs and EZH2 is thought to modulate gene expression and responses to retinoic acid signalling. PRAME also interacts with the outer membrane opacity protein (OPA-P) from bacterial pathogen *N. gonorrhoea*, and may also interact with other pathogen-associated microbial patterns (PAMPs) entering the cytoplasm. In addition, expression of PRAME in cancer cells may allow it to function in sensing molecules associated with cancer or cancer-related inflammation.

Human PRAME and its paralogues are related to LRR family proteins, some of which are known to have functions in cell immunity and signal transduction. PRAME may therefore serve as an intracellular sensor of pathogen associated molecular particles (PAMPs) or molecules associated with cancer-related inflammation (Fig. 3). To confirm this, it will be important to identify proteins or other molecules that associate with PRAME, and determine whether PRAME adopts a structure similar to the LRR domains of TLR or NALP proteins. Murine orthologues of PRAME show an expression pattern that is restricted to the zygote, and later to ovary and testis tissues, suggesting that this family of proteins also functions in early embryogenesis and gametogenesis. PRAME expression in cancers may therefore be due to reactivation of genes associated with 'stemness' or pluripotency, or in response to signals that activate immune or autoimmune responses associated with tumours.

The subcellular distribution of PRAME indicates it is likely to have both nuclear and cytoplasmic functions. Although direct interactions with retinoic acid receptors appear weak (Fig. 2), there is substantial evidence that PRAME may negatively regulate retinoic acid signalling, through recruitment of polycomb proteins such as EZH2 to promoter complexes (Fig. 3). It remains to be determined how these molecular functions of PRAME exert their effects on differentiation and proliferation of leukaemic cells. Thus, while the precise molecular functions of PRAME and its role in oncogenesis remain to be addressed, PRAME continues to serve as both a useful prognostic marker in acute leukaemias and solid tumours, and an attractive target for potential immunotherapy.

## Abbreviations

PRAME: preferentially expressed antigen in melanoma; MAPE: melanoma antigen preferentially expressed in tumours; OIP4: opa interacting protein; LRR: leucine-rich repeat; HLA: human leucocyte antigen; BCR: breakpoint cluster region; qPCR: semi-quantitative polymerase chain reaction; CML: chronic myeloid leukaemia; CTA: cancer testis antigen; 5'UTR: 5' untranslated region; AML: acute myeloid leukaemia; ALL: acute lymphoblastic leukaemia; NALP: NACHT-, LRR-and PYD-containing proteins; TLR: toll-like receptor; PAMP: pathogen-associated molecular patterns; GFP: green fluorescent protein; NLS: nuclear localisation signal; RA: retinoic acid; RAR: retinoic acid receptor; HDAC: histone deacetylase; TAP: tandem affinity purification; LBD: ligand binding domain; GST: glutathione S-transferase; ER: oestrogen receptor; RXR: 9-*cis *retinoic acid receptor; SRC1: steroid receptor co-activator; HDACi: histone deacetylase inhibitors; ATRA: all-*trans *retinoic acid; MRD: minimal residual disease.

## Competing interests

The authors declare that they have no competing interests.

## Authors' contributions

FW and DMH reviewed the literature, and wrote and edited the manuscript. FW, KS and JF generated experimental data. PM, JE and DMH performed sequence alignments, motif searches and structure predictions. All authors read and approved the final manuscript.

## References

[B1] Ikeda H Lethe B Lehmann F van Baren N Baurain JF de Smet C Chambost H Vitale M Moretta A Boon T Coulie PG Characterization of an antigen that is recognized on a melanoma showing partial HLA loss by CTL expressing an NK inhibitory receptor Immunity 1997 6 199 208 10.1016/S1074-7613(00)80426-4 9047241

[B2] Haqq C Nosrati M Sudilovsky D Crothers J Khodabakhsh D Pulliam BL Federman S Miller JR Allen RE Singer MI The gene expression signatures of melanoma progression Proc Natl Acad Sci USA 2005 102 6092 6097 10.1073/pnas.0501564102 15833814 PMC1087936

[B3] Williams JM Chen GC Zhu L Rest RF Using the yeast two-hybrid system to identify human epithelial cell proteins that bind gonococcal Opa proteins: intracellular gonococci bind pyruvate kinase via their Opa proteins and require host pyruvate for growth Mol Microbiol 1998 27 171 186 10.1046/j.1365-2958.1998.00670.x 9466265

[B4] Nakamura Y Tanaka F Nagahara H Ieta K Haraguchi N Mimori K Sasaki A Inoue H Yanaga K Mori M Opa interacting protein 5 (OIP5) is a novel cancer-testis specific gene in gastric cancer Ann Surg Oncol 2007 14 885 892 10.1245/s10434-006-9121-x 17151793

[B5] Kawasaki K Minoshima S Nakato E Shibuya K Shintani A Schmeits JL Wang J Shimizu N One-megabase sequence analysis of the human immunoglobulin lambda gene locus Genome Res 1997 7 250 261 10.1101/gr.7.3.250 9074928

[B6] Croce CM Huebner K Isobe M Fainstain E Lifshitz B Shtivelman E Canaani E Mapping of four distinct BCR-related loci to chromosome region 22q11: order of BCR loci relative to chronic myelogenous leukemia and acute lymphoblastic leukemia breakpoints Proc Natl Acad Sci USA 1987 84 7174 7178 10.1073/pnas.84.20.7174 3118359 PMC299252

[B7] Schenk T Stengel S Goellner S Steinbach D Saluz HP Hypomethylation of PRAME is responsible for its aberrant overexpression in human malignancies Genes Chromosomes Cancer 2007 46 796 804 10.1002/gcc.20465 17534929

[B8] Epping MT Wang L Edel MJ Carlee L Hernandez M Bernards R The human tumor antigen PRAME is a dominant repressor of retinoic acid receptor signaling Cell 2005 122 835 847 10.1016/j.cell.2005.07.003 16179254

[B9] Quintarelli C Dotti G De Angelis B Hoyos V Mims M Luciano L Heslop HE Rooney CM Pane F Savoldo B Cytotoxic T lymphocytes directed to the preferentially expressed antigen of melanoma (PRAME) target chronic myeloid leukemia Blood 2008 112 1876 1885 10.1182/blood-2008-04-150045 18591381 PMC3401035

[B10] Partheen K Levan K Osterberg L Claesson I Fallenius G Sundfeldt K Horvath G Four potential biomarkers as prognostic factors in stage III serous ovarian adenocarcinomas Int J Cancer 2008 123 2130 2137 10.1002/ijc.23758 18709641

[B11] Proto-Siqueira R Figueiredo-Pontes LL Panepucci RA Garcia AB Rizzatti EG Nascimento FM Ishikawa HC Larson RE Falcao RP Simpson AJ PRAME is a membrane and cytoplasmic protein aberrantly expressed in chronic lymphocytic leukemia and mantle cell lymphoma Leuk Res 2006 30 1333 1339 10.1016/j.leukres.2006.02.031 16620968

[B12] Radich JP Dai H Mao M Oehler V Schelter J Druker B Sawyers C Shah N Stock W Willman CL Gene expression changes associated with progression and response in chronic myeloid leukemia Proc Natl Acad Sci USA 2006 103 2794 2799 10.1073/pnas.0510423103 16477019 PMC1413797

[B13] van Baren N Chambost H Ferrant A Michaux L Ikeda H Millard I Olive D Boon T Coulie PG PRAME, a gene encoding an antigen recognized on a human melanoma by cytolytic T cells, is expressed in acute leukaemia cells Br J Haematol 1998 102 1376 1379 10.1046/j.1365-2141.1998.00982.x 9753074

[B14] Steinbach D Hermann J Viehmann S Zintl F Gruhn B Clinical implications of PRAME gene expression in childhood acute myeloid leukemia Cancer Genet Cytogenet 2002 133 118 123 10.1016/S0165-4608(01)00570-2 11943337

[B15] Oehler VG Guthrie KA Cummings CL Sabo K Wood BL Gooley T Yang T Epping MT Shou Y Pogosova-Agadjanyan E The preferentially expressed antigen in melanoma (PRAME) inhibits myeloid differentiation in normal hematopoietic and leukemic progenitor cells Blood 2009 114 3299 3308 10.1182/blood-2008-07-170282 19625708 PMC2759652

[B16] Santamaria C Chillon MC Garcia-Sanz R Balanzategui A Sarasquete ME Alcoceba M Ramos F Bernal T Queizan JA Penarrubia MJ The relevance of preferentially expressed antigen of melanoma (PRAME) as a marker of disease activity and prognosis in acute promyelocytic leukemia Haematologica 2008 93 1797 1805 10.3324/haematol.13214 18815192

[B17] Qin Y Zhu H Jiang B Li J Lu X Li L Ruan G Liu Y Chen S Huang X Expression patterns of WT1 and PRAME in acute myeloid leukemia patients and their usefulness for monitoring minimal residual disease Leuk Res 2009 33 384 390 10.1016/j.leukres.2008.08.026 18950857

[B18] Matsushita M Ikeda H Kizaki M Okamoto S Ogasawara M Ikeda Y Kawakami Y Quantitative monitoring of the PRAME gene for the detection of minimal residual disease in leukaemia Br J Haematol 2001 112 916 926 10.1046/j.1365-2141.2001.02670.x 11298586

[B19] Steinbach D Viehmann S Zintl F Gruhn B PRAME gene expression in childhood acute lymphoblastic leukemia Cancer Genet Cytogenet 2002 138 89 91 10.1016/S0165-4608(02)00582-4 12419593

[B20] Proto-Siqueira R Falcao RP de Souza CA Ismael SJ Zago MA The expression of PRAME in chronic lymphoproliferative disorders Leuk Res 2003 27 393 396 10.1016/S0145-2126(02)00217-5 12620290

[B21] Pellat-Deceunynck C Mellerin MP Labarriere N Jego G Moreau-Aubry A Harousseau JL Jotereau F Bataille R The cancer germ-line genes MAGE-1, MAGE-3 and PRAME are commonly expressed by human myeloma cells Eur J Immunol 2000 30 803 809 10.1002/1521-4141(200003)30:3<803::AID-IMMU803>3.0.CO;2-P 10741395

[B22] Andrade VC Vettore AL Felix RS Almeida MS Carvalho F Oliveira JS Chauffaille ML Andriolo A Caballero OL Zago MA Colleoni GW Prognostic impact of cancer/testis antigen expression in advanced stage multiple myeloma patients Cancer Immun 2008 8 2 18237105 PMC2935785

[B23] Doolan P Clynes M Kennedy S Mehta JP Crown J O'Driscoll L Prevalence and prognostic and predictive relevance of PRAME in breast cancer Breast Cancer Res Treat 2008 109 359 365 10.1007/s10549-007-9643-3 17624586

[B24] Epping MT Hart AA Glas AM Krijgsman O Bernards R PRAME expression and clinical outcome of breast cancer Br J Cancer 2008 99 398 403 10.1038/sj.bjc.6604494 18648365 PMC2527791

[B25] Sun Y Urquidi V Goodison S Derivation of molecular signatures for breast cancer recurrence prediction using a two-way validation approach Breast Cancer Res Treat 2009 119 3 593 9 10.1007/s10549-009-0365-6 19291396 PMC2844120

[B26] Figueiredo DL Mamede RC Proto-Siqueira R Neder L Silva WA Jr Zago MA Expression of cancer testis antigens in head and neck squamous cell carcinomas Head Neck 2006 28 614 619 10.1002/hed.20380 16475205

[B27] Boon K Edwards JB Siu IM Olschner D Eberhart CG Marra MA Strausberg RL Riggins GJ Comparison of medulloblastoma and normal neural transcriptomes identifies a restricted set of activated genes Oncogene 2003 22 7687 7694 10.1038/sj.onc.1207043 14576832

[B28] Oberthuer A Hero B Spitz R Berthold F Fischer M The tumor-associated antigen PRAME is universally expressed in high-stage neuroblastoma and associated with poor outcome Clin Cancer Res 2004 10 4307 4313 10.1158/1078-0432.CCR-03-0813 15240516

[B29] Watari K Tojo A Nagamura-Inoue T Nagamura F Takeshita A Fukushima T Motoji T Tani K Asano S Identification of a melanoma antigen, PRAME, as a BCR/ABL-inducible gene FEBS Lett 2000 466 367 371 10.1016/S0014-5793(00)01112-1 10682862

[B30] Passeron T Valencia JC Namiki T Vieira WD Passeron H Miyamura Y Hearing VJ Upregulation of SOX9 inhibits the growth of human and mouse melanomas and restores their sensitivity to retinoic acid J Clin Invest 2009 119 954 963 19273910 10.1172/JCI34015 PMC2662541

[B31] De Smet C De Backer O Faraoni I Lurquin C Brasseur F Boon T The activation of human gene MAGE-1 in tumor cells is correlated with genome-wide demethylation Proc Natl Acad Sci USA 1996 93 7149 7153 10.1073/pnas.93.14.7149 8692960 PMC38951

[B32] Sigalotti L Fratta E Coral S Tanzarella S Danielli R Colizzi F Fonsatti E Traversari C Altomonte M Maio M Intratumor heterogeneity of cancer/testis antigens expression in human cutaneous melanoma is methylation-regulated and functionally reverted by 5-aza-2'-deoxycytidine Cancer Res 2004 64 9167 9171 10.1158/0008-5472.CAN-04-1442 15604288

[B33] Ortmann CA Eisele L Nuckel H Klein-Hitpass L Fuhrer A Duhrsen U Zeschnigk M Aberrant hypomethylation of the cancer-testis antigen PRAME correlates with PRAME expression in acute myeloid leukemia Ann Hematol 2008 87 809 818 10.1007/s00277-008-0514-8 18587578

[B34] Roman-Gomez J Jimenez-Velasco A Agirre X Castillejo JA Navarro G Jose-Eneriz ES Garate L Cordeu L Cervantes F Prosper F Epigenetic regulation of PRAME gene in chronic myeloid leukemia Leuk Res 2007 31 1521 1528 10.1016/j.leukres.2007.02.016 17382387

[B35] Luetkens T Schafhausen P Uhlich F Stasche T Akbulak R Bartels BM Hildebrandt Y Gontarewicz A Kobold S Meyer S Expression, epigenetic regulation, and humoral immunogenicity of cancer-testis antigens in chronic myeloid leukemia Leuk Res 2010 in press 20409582 10.1016/j.leukres.2010.03.039

[B36] Gunn SR Bolla AR Barron LL Gorre ME Mohammed MS Bahler DW Mellink CH van Oers MH Keating MJ Ferrajoli A Array CGH analysis of chronic lymphocytic leukemia reveals frequent cryptic monoallelic and biallelic deletions of chromosome 22q11 that include the PRAME gene Leuk Res 2009 33 1276 1281 10.1016/j.leukres.2008.10.010 19027161

[B37] Bea S Salaverria I Armengol L Pinyol M Fernandez V Hartmann EM Jares P Amador V Hernandez L Navarro A Uniparental disomies, homozygous deletions, amplifications, and target genes in mantle cell lymphoma revealed by integrative high-resolution whole-genome profiling Blood 2009 113 3059 3069 10.1182/blood-2008-07-170183 18984860 PMC2662646

[B38] Birtle Z Goodstadt L Ponting C Duplication and positive selection among hominin-specific PRAME genes BMC Genomics 2005 6 120 10.1186/1471-2164-6-120 16159394 PMC1262708

[B39] Tian X Pascal G Monget P Evolution and functional divergence of NLRP genes in mammalian reproductive systems BMC Evol Biol 2009 9 202 10.1186/1471-2148-9-202 19682372 PMC2735741

[B40] Evsikov AV Graber JH Brockman JM Hampl A Holbrook AE Singh P Eppig JJ Solter D Knowles BB Cracking the egg: molecular dynamics and evolutionary aspects of the transition from the fully grown oocyte to embryo Genes Dev 2006 20 2713 2727 10.1101/gad.1471006 17015433 PMC1578697

[B41] Dade S Callebaut I Mermillod P Monget P Identification of a new expanding family of genes characterized by atypical LRR domains. Localization of a cluster preferentially expressed in oocyte FEBS Lett 2003 555 533 538 10.1016/S0014-5793(03)01341-3 14675769

[B42] Kajava AV Structural diversity of leucine-rich repeat proteins J Mol Biol 1998 277 519 527 10.1006/jmbi.1998.1643 9533877

[B43] McEwan PA Scott PG Bishop PN Bella J Structural correlations in the family of small leucine-rich repeat proteins and proteoglycans J Struct Biol 2006 155 294 305 10.1016/j.jsb.2006.01.016 16884925

[B44] Kobe B Deisenhofer J Crystal structure of porcine ribonuclease inhibitor, a protein with leucine-rich repeats Nature 1993 366 751 756 10.1038/366751a0 8264799

[B45] Kelley LA Sternberg MJ Protein structure prediction on the Web: a case study using the Phyre server Nat Protoc 2009 4 363 371 10.1038/nprot.2009.2 19247286

[B46] Lee MS Kim YJ Signaling pathways downstream of pattern-recognition receptors and their cross talk Annu Rev Biochem 2007 76 447 480 10.1146/annurev.biochem.76.060605.122847 17328678

[B47] Tajeddine N Gala JL Louis M Van Schoor M Tombal B Gailly P Tumor-associated antigen preferentially expressed antigen of melanoma (PRAME) induces caspase-independent cell death in vitro and reduces tumorigenicity in vivo Cancer Res 2005 65 7348 7355 10.1158/0008-5472.CAN-04-4011 16103086

[B48] Epping MT Wang L Plumb JA Lieb M Gronemeyer H Brown R Bernards R A functional genetic screen identifies retinoic acid signaling as a target of histone deacetylase inhibitors Proc Natl Acad Sci USA 2007 104 17777 17782 10.1073/pnas.0702518104 17968018 PMC2077016

[B49] Heery DM Kalkhoven E Hoare S Parker MG A signature motif in transcriptional co-activators mediates binding to nuclear receptors Nature 1997 387 733 736 10.1038/42750 9192902

[B50] Lehner B Semple JI Brown SE Counsell D Campbell RD Sanderson CM Analysis of a high-throughput yeast two-hybrid system and its use to predict the function of intracellular proteins encoded within the human MHC class III region Genomics 2004 83 153 167 10.1016/S0888-7543(03)00235-0 14667819

[B51] Markson G Kiel C Hyde R Brown S Charalabous P Bremm A Semple J Woodsmith J Duley S Salehi-Ashtiani K Analysis of the human E2 ubiquitin conjugating enzyme protein interaction network Genome Res 2009 19 1905 1911 10.1101/gr.093963.109 19549727 PMC2765280

[B52] Steinbach D Pfaffendorf N Wittig S Gruhn B PRAME expression is not associated with down-regulation of retinoic acid signaling in primary acute myeloid leukemia Cancer Genet Cytogenet 2007 177 51 54 10.1016/j.cancergencyto.2007.05.011 17693191

[B53] Goellner S Steinbach D Schenk T Gruhn B Zintl F Ramsay E Saluz HP Childhood acute myelogenous leukaemia: association between PRAME, apoptosis-and MDR-related gene expression Eur J Cancer 2006 42 2807 2814 10.1016/j.ejca.2006.06.018 16978861

[B54] Greiner J Schmitt M Li L Giannopoulos K Bosch K Schmitt A Dohner K Schlenk RF Pollack JR Dohner H Bullinger L Expression of tumor-associated antigens in acute myeloid leukemia: Implications for specific immunotherapeutic approaches Blood 2006 108 4109 4117 10.1182/blood-2006-01-023127 16931630

[B55] Tajeddine N Millard I Gailly P Gala JL Real-time RT-PCR quantification of PRAME gene expression for monitoring minimal residual disease in acute myeloblastic leukaemia Clin Chem Lab Med 2006 44 548 555 10.1515/CCLM.2006.106 16681423

[B56] Slovak ML Kopecky KJ Cassileth PA Harrington DH Theil KS Mohamed A Paietta E Willman CL Head DR Rowe JM Karyotypic analysis predicts outcome of preremission and postremission therapy in adult acute myeloid leukemia: a Southwest Oncology Group/Eastern Cooperative Oncology Group Study Blood 2000 96 4075 4083 11110676

[B57] Santamaria CM Chillon MC Garcia-Sanz R Perez C Caballero MD Ramos F Garcia de Coca A Alonso JM Giraldo P Bernal T Molecular stratification model for prognosis in cytogenetically normal acute myeloid leukemia (CN-AML) Blood 2009 114 148 152 10.1182/blood-2008-11-187724 19398719

[B58] Kawano R Karube K Kikuchi M Takeshita M Tamura K Uike N Eto T Ohshima K Suzumiya J Oncogene associated cDNA microarray analysis shows PRAME gene expression is a marker for response to anthracycline containing chemotherapy in patients with diffuse large B-cell lymphoma J Clin Exp Hematop 2009 49 1 7 10.3960/jslrt.49.1 19474511

[B59] Staege MS Banning-Eichenseer U Weissflog G Volkmer I Burdach S Richter G Mauz-Korholz C Foll J Korholz D Gene expression profiles of Hodgkin's lymphoma cell lines with different sensitivity to cytotoxic drugs Exp Hematol 2008 36 886 896 10.1016/j.exphem.2008.02.014 18400362

[B60] Steinbach D Schramm A Eggert A Onda M Dawczynski K Rump A Pastan I Wittig S Pfaffendorf N Voigt A Identification of a set of seven genes for the monitoring of minimal residual disease in pediatric acute myeloid leukemia Clin Cancer Res 2006 12 2434 2441 10.1158/1078-0432.CCR-05-2552 16638849

[B61] Paydas S Tanriverdi K Yavuz S Seydaoglu G PRAME mRNA levels in cases with chronic leukemia: Clinical importance and review of the literature Leuk Res 2007 31 365 369 10.1016/j.leukres.2006.06.022 16914202

[B62] Greiner J Dohner H Schmitt M Cancer vaccines for patients with acute myeloid leukemia--definition of leukemia-associated antigens and current clinical protocols targeting these antigens Haematologica 2006 91 1653 1661 17145602

[B63] Griffioen M Kessler JH Borghi M van Soest RA van der Minne CE Nouta J van der Burg SH Medema JP Schrier PI Falkenburg JH Detection and functional analysis of CD8+ T cells specific for PRAME: a target for T-cell therapy Clin Cancer Res 2006 12 3130 3136 10.1158/1078-0432.CCR-05-2578 16707612

[B64] Rezvani K Yong AS Tawab A Jafarpour B Eniafe R Mielke S Savani BN Keyvanfar K Li Y Kurlander R Barrett AJ Ex vivo characterization of polyclonal memory CD8+ T-cell responses to PRAME-specific peptides in patients with acute lymphoblastic leukemia and acute and chronic myeloid leukemia Blood 2009 113 2245 2255 10.1182/blood-2008-03-144071 18988867 PMC2652370

[B65] Greiner J Ringhoffer M Taniguchi M Li L Schmitt A Shiku H Dohner H Schmitt M mRNA expression of leukemia-associated antigens in patients with acute myeloid leukemia for the development of specific immunotherapies Int J Cancer 2004 108 704 711 10.1002/ijc.11623 14696097

[B66] Greiner J Bullinger L Guinn BA Dohner H Schmitt M Leukemia-associated antigens are critical for the proliferation of acute myeloid leukemia cells Clin Cancer Res 2008 14 7161 7166 10.1158/1078-0432.CCR-08-1102 19010831

[B67] Zhang Y Shahriar M Zhang J Ahmed SU Lim SH Clofarabine induces hypomethylation of DNA and expression of Cancer-Testis antigens Leuk Res 2009 33 1678 1683 10.1016/j.leukres.2009.04.005 19427036

[B68] Heery DM Hoare S Hussain S Parker MG Sheppard H Core LXXLL motif sequences in CREB-binding protein, SRC1, and RIP140 define affinity and selectivity for steroid and retinoid receptors J Biol Chem 2001 276 6695 6702 10.1074/jbc.M009404200 11078741

[B69] Coulthard VH Matsuda S Heery DM An extended LXXLL motif sequence determines the nuclear receptor binding specificity of TRAP220 J Biol Chem 2003 278 10942 10951 10.1074/jbc.M212950200 12556447

